# An unusual case of anisocoria by vegetal intoxication: a case report

**DOI:** 10.1186/1824-7288-36-50

**Published:** 2010-07-20

**Authors:** Marina Macchiaiolo, Elettra Vignati, Michaela V Gonfiantini, Annalisa Grandin, Maria Teresa Romano, Michele Salata, Diletta Valentini, Alberto Villani

**Affiliations:** 1 U.O.C Pediatria Generale Dipartimento di Medicina Pediatrica, Ospedale Pediatrico Bambino Gesù, P.zza Sant'Onofrio 4, 00165 Roma, Italy

## Abstract

A 12 year old boy presented with an acute onset of anisocoria and blurred vision. Ocular motility was normal but his right pupil was dilated, round but sluggishly reactive to light. There was no history of trauma, eye drops' instillation, nebulised drugs or local ointments. His past medical history was negative.

A third nerve palsy was considered but the performed cerebral MRI was normal.

On further anamnestic investigation the boy revealed that he had spent the morning doing gardening, and especially working on a "trumpet plant". *Datura *and *Brugmansia *are well known toxic plant; all *Datura *and *Brugmasia *plants contain, primarily in their seeds and flowers, tropane alkaloids such as scopolamine, hyoscyamine and atropine. Systemic and local intoxications have already been described.

The day after anisocoria was much less evident and completely resolved in three days.

We present this case of an unusual cause of mydriasis to underline once more the importance of a well and deeply conducted medical history.

## Case report

**Figure 1:**
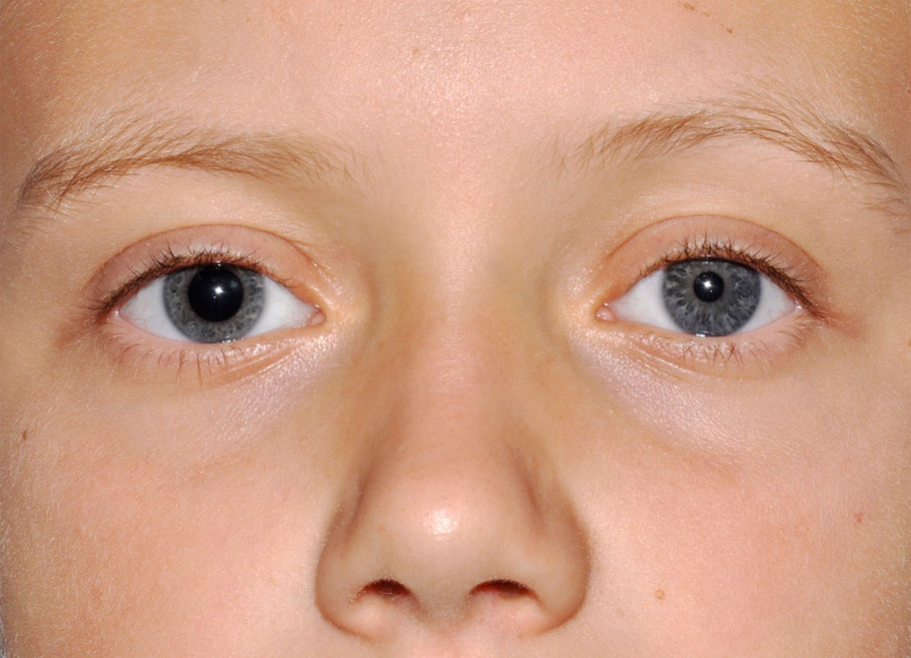
**Evident right mydriasis**.

A 12 year old boy presented to the emergency for the onset of acute anisocoria. Two hours before the boy had started to complain of a blurred vision; his mother, a paediatric nurse, noticed a pupil asymmetry with a clearly dilated right pupil (Figure 1). Eyes were normal at wake up and during the early morning; the boy had spent the all morning playing in grandmother's garden. There was no history of trauma, eye drops' instillation, nebulised drugs or local ointments. His past medical history was negative.

On physical examination the boy was well, orientated and collaborative. In room light the right pupil was round, but the reaction to light was sluggish. Left eye's reaction was normal. Anisocoria was more evident in bright light. Ocular motility was normal. On slit lamp the pupil was round and no signs of trauma were detected. Consensual pupillary light reflex of the left eye was elicited. In the anterior chambers no inflammatory changes were detected and both irises and fundi examination were normal.

A third nerve palsy was considered but the performed cerebral MRI was normal.

**Figure 2:**
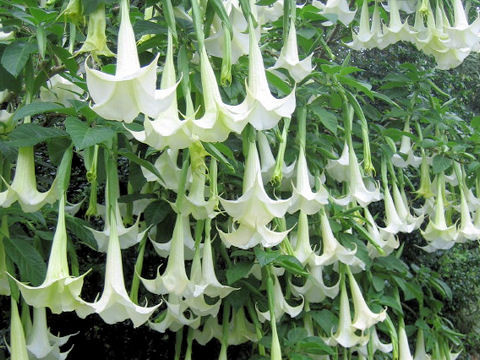
***Datura suaveolens***.

On further investigation the boy explained that he had spent the morning doing gardening and especially working on a "trumpet plant". A quick internet search helped to find a picture of *" Datura suaveolens *", commonly known as trumpet plant, immediately recognized by the child and his mother (Figure 2). The boy did not refer a direct contact of leaves or flowers with eyes, however he referred to have handled both leaves and flowers and to have removed new small plants arising at the bottom of the plant. It is very likely that an accidental hands-eye contact after touching the plant had occurred.

The day after anisocoria was much less evident and completely resolved in three days. The patient was diagnosed with pharmacologic mydriasis secondary to unintentional contact with *Datura suaveolens *; no further investigations were performed and the boy completely recovered.

## Discussion

Causes of a mydriasis, more evident in light, are trauma, third nerve palsy, Adie's pupil and pharmacologic mydriasis [1].

Our patient had no neurologic symptoms, there was no ptosis nor an ocular motility deficit. However, a cerebral MRI was performed to exclude a compressive origin of palsy even if the level of suspicion of this cause was very low.

An Adie's pupil due to an injury to the ciliary ganglion can be secondary to viral infection, trauma or cancer. To differentiate it from other causes of mydriasis a pilocoarpine test with low concentration (0.1%) can be useful. Pilocarpine will cause constriction of the pupil due to denervation sensitivity, while no constriction is detected in case of oculomotor palsy or pharmacological mydriasis.

Differentiation between paralytic and pharmacologic mydriasis can be made by using a 1% pilocarpine eye drops test. Dilated pupils do not constrict in response to pilocarpine 1% in pharmacologic mydriasis, but do in paralytic mydriasis. Also mydriasis secondary to a traumatism does not respond to drops, but in this case there was no history nor any objective sign of trauma on slit lamp examination [2]. Furthermore this test may not always be reliable. In the case of pharmacological mydriasis due to an uncontrolled toxicity exposure, the concentration of alkaloids is unpredictable; if the degree of toxicity is low, mydriasis may be present but it can be overcame with pilocarpine drops.

In this case, a detailed medical history helped to final diagnosis of pharmacologic mydriasis by revealing the exposure to an ornamental plant, called angel's trumpet, frequently found in gardens. Accidental mydriasis from exposure to plant components has been reported in humans and animals [2]. *Brugmansia *is a genus of seven species of flowering plants of the family Solanaceae native from subtropical regions of South America. They are known as angel's trumpets, sharing that name with the closely related genus *Datura*, well known toxic plants of the Solanaceae family.

All *Brugmansia *and *Datura *plants contain, primarily in their seeds and flowers, tropane alkaloids such as scopolamine, hyoscyamine and atropine. Because of the presence of such substances, these plants have been used for centuries in some cultures as poison and hallucinogen [2,3].

Flowers that can induce parasympatholytic effects in the eye are well known: deadly nightshade (Atropa belladonna), angel's trumpet (Brugmansia arborea), thorn apple or jimsonweed (Datura stramonium), and black henbane (Hyoscyamus niger).

The Brugmansia genus (angel's trumpet) contains a concentration of tropane alkaloids ranging from 2,5 mg/g to 7 mg/g per blossom, 0.20 mg of atropine and 0.65 mg of scopolamine [3]. In Datura (thorn apple) the seeds have the highest alkaloid content of the plant: 2.71 mg/g of atropine and 0.66 mg/g of scopolamine; leaves of the thorn apple contain 0.2 to 0.45 mg/g of total alkaloids. The alkaloid concentration is always referred to grams of fresh plant weight.

Andreola et al (2008) reported a very similar case [4], with rapid onset of symptoms after exposure; authors have analysed alkaloid content in the different parts of the flower. Their analysis showed that the maximum alkaloid content is present in the corolla tissue along the ribs; authors [4] remark that the abundant occurrence of glandular hairs on the rib regions suggests that these alkaloids might be present not only in the epidermal cells but also in the glandular hairs explaining why intoxication may occur, following simple contact of the eye with the plant surface, which is characterized by high densities of glandular hairs that can be easily broken.

However alkaloid concentration in leaves, stems, flowers and seeds of an individual plant differ markedly; it even changes with season and hydration.

That's why it is almost impossible to determine the exact dose of alkaloids in the plant component that comes in contact with the eye.

Ingestion of 10 to 50 mg of atropine appears to be toxic for adults, 4 to 5 mg may be fatal for small children. The duration of the mydriasis after exposure to a plant component varies from 24 hours to 1 week. The presentation of the mydriasis can differ from a maximally dilated and non-responsive pupil to variable degrees of light responses. Systemic intoxication by ingestion has been widely described. Symptoms of *Datura *toxicity occur typically within 60 minutes after ingestion and continue for 24-48 hours. Ingestion of *Datura *manifests as a classic anticholinergic syndrome comprising central and peripheral signs and symptoms [5,6].

By help of the well conducted anamnesis our patient was diagnosed with pharmacologic mydriasis secondary to unintentional contact with *Datura suaveolens *; no further investigations were performed and the boy completely recovered.

## Conclusions

This case highlights once more the importance of a well conducted medical history Suspicion of an accidental intoxication should always arise when unilateral mydriasis is found in patients without evidence of a retrobulbar or intracerebral process. Questions about playing or working in the garden should always be asked. We performed an instrumental exam like cerebral MRI, with an earlier and deeper anamnestic investigation this exam could have been avoided.

## Consent

Written informed consent was obtained from the patient's parents for publication of this case report and for the use of image in this case report. A copy of the written consent is available for review by the Editor-in-Chief of this journal.

## Competing interests

The authors declare that they have no competing interests.

## Authors' contributions

All authors have participated in the diagnostic pathways, all authors have read and approved he final manuscript.
